# ﻿Taxonomic novelties in Dictyosporiaceae and Pleurotremataceae (Dothideomycetes, Ascomycota): Two new species and three new host reports in the coastal region of Guangdong Province, China

**DOI:** 10.3897/mycokeys.125.171612

**Published:** 2025-11-17

**Authors:** Nimali I. de Silva, Danushka S. Tennakoon, Ning Xie, Sinang Hongsanan

**Affiliations:** 1 Shenzhen Key Laboratory of Microbial Genetic Engineering, College of Life Science and Oceanography, Shenzhen University, Shenzhen 518060, China Shenzhen University Shenzhen China; 2 Guangdong Provincial Key Laboratory for Plant Epigenetics, College of Life Sciences and Oceanography, Shenzhen University, Shenzhen 518060, China Shenzhen University Shenzhen China

**Keywords:** Dyfrolomycetales, multi-gene phylogeny, Pleosporales, saprobes, systematics, taxonomy

## Abstract

During a survey of microflora associated with dead plant substrates in coastal regions of Guangdong Province, China, we identified several interesting Dothideomycetes fungi and provided refined updated phylogenetic analyses for *Dictyosporium* and *Melomastia* species. Two novel species are introduced, *Dictyosporium thecatum* and *Melomastia shenzhenensis*, based on molecular and morphological evidence. New host records of *M. beihaiensis*, *M. hydei* and *M. fusispora* are also reported in this paper. Molecular phylogenetic studies of new isolates were based on concatenated (i) ITS, LSU, *tef*1-α and (ii) LSU, SSU, ITS, *tef*1-α gene regions for *Dictyosporium* and *Melomastia*, respectively. These taxonomic novelties were recognised by comparing morphological characteristics with closely-related taxa. *Melomastia shenzhenensis* closely clustered with *M. oleae* (CGMCC 3.20619 and UESTCC 21.0003) with high statistical support value (ML/BYPP = 100%/1.00). Morphologically, *Melomastia shenzhenensis* can be distinguished from *M. oleae* in having larger of ascomata, ostiolar canal and peridium and smaller asci and ascospores. *Dictyosporium thecatum* forms a distinct basal clade with *D. sexualis* (MFLUCC 10-0127). *Dictyosporium thecatum* is different from *D. sexualis* in having smaller ascomata, asci and ascospores and possesses a larger mucilaginous sheath surrounding ascospores than *D. sexualis*. Two pairwise identity analyses were conducted for *Dictyosporium* and *Melomastia*. The resulting sequence identity scores were saved as a matrix and visualised as plots with a colour key to indicate the correspondence between pairwise identities. This study offers new insights into saprobic Dothideomycetes colonising dead woody substrates in coastal habitats of Guangdong Province, China.

## ﻿Introduction

Coastal regions encompass some of the most biodiverse and unique ecosystems on Earth. These maritime zones are home to the oceans’ bounty that encompass a broad range of habitat types, including coral reefs, kelp forests, seagrass, tidal flats, mangroves, estuaries, salt marshes, wetlands and coastal wooded habitat (Williams et al. 2022). China is one of the countries possessing numerous sea resources with a vast maritime region (Ren et al. 2018). The persistence of many fungal species relies upon diverse macro and micro habitats in coastal regions. Despite the availability of abundant ecological niches, research on the fungi inhabiting coastal south China region remains limited. Consequently, exploration of fungi in poorly-understood geographical locations, such as maritime ecological regions of China, would probably contribute to reveal novel taxa and expand host and geographical association of known species.

The South China Sea is the largest marginal sea in the western Pacific, covering ~ 3.5 million km^2^ along the continental margin of East Asia and averaging over 2,000 m in depth (Morton and Blackmore 2001; Yao and Wang 2021; Wang et al. 2022). Since it is a marginal sea, it is surrounded by extensive landmasses (Morton and Blackmore 2001). The South China Sea lies at the centre of the Indo-West Pacific biogeographic region harbouring the world’s most diverse shallow-water maritime ocean (Morton and Blackmore 2001). The south China coastline harbours different ecosystem types, i.e. coral reefs, seagrasses, mangroves, wetlands, bays and coastal vegetation (Morton and Blackmore 2001; Zhang et al. 2025). The largest mainland coastline in the South China Sea can be seen in Guangdong Province which spans along 14 coastal cities (Zhang et al. 2025). The Shenzhen Bay Estuary is located on the east coast of the Pearl River Estuary in South China, in Guangdong Province (Xu et al. 2022). Eight rivers, including the Fengtang River, flow into Shenzhen Bay (Xu et al. 2022) indicating an ecologically valuable habitat for fungal identification. The natural vegetation in Shenzhen Bay consisting of mangroves, semi-mangroves, wetland plants and terrestrial plants, was restored from the bund to the inner bank of the Fengtang River (Xu et al. 2022). These unique geographical locations provide a suitable growth environment for abundant fungal resources. In addition, Guangdong Province has a rich diversity of plants, ranking it sixth in China (Zhuqiu et al. 2023). The vegetation comprises a variety of plants, including native wild higher plants, bryophytes, lycopods and pteridophytes, gymnosperms and angiosperms (Zhuqiu et al. 2023). The most common and widely distributed angiosperms are Poaceae, Fabaceae, Orchidaceae, Cyperaceae, Rubiaceae, Lamiaceae, Asteraceae, Rosaceae, Lauraceae and Gesneriaceae (Zhuqiu et al. 2023). Apart from that, the southern China coastal region is considered as one of the major mangrove regions in the world (Luo et al. 2022). Further, the fungal resources in this region have been exploited and utilised to research secondary metabolites, such as anti-tumour drugs to treat cancer (Luo et al. 2022).

Dothideomycetes represent a major taxonomic group of Ascomycota, encompassing more than 25 orders, 110 families and over 19,000 species (Wijayawardene et al. 2022). The species of this group are characterised by bitunicate asci, typically with fissitunicate dehiscence and many species have been collected from freshwater habitats (Dong et al. 2020; Pem et al. 2021; Wang et al. 2025). They show cosmopolitan distribution and occur in a wide variety of environments including terrestrial, marine, freshwater and extreme environments (Suetrong et al. 2009; Dayarathne et al. 2020; Dong et al. 2020; Pem et al. 2021). Terrestrial fungi occur in a variety of substrata, soil, rocks and in plants and exhibit different life-styles as saprobes, plant pathogens, endophytes, epiphytes, ectomycorrhizal, lichens, lichenicolous, nematode-trapping and rock-inhabiting members (Taylor and Sinsabaugh 2015; Hongsanan et al. 2020; Pem et al. 2021; Wanasinghe et al. 2022). In terms of marine mycota, fungi associated with plants in coastal region receive little attention compared to mangrove ecosystems. Therefore, our aim is to investigate and identify some of the saprobic Dothideomycetes fungi in Shenzhen Bay region in Guandong Province, China. In this study, we mainly focus on *Dictyosporium* species (Dictyosporiaceae, Pleosporales) and *Melomastia* species (Pleurotremataceae, Dyfrolomycetales, Dothideomycetes orders *incertae sedis*). The overall intention of this study has been to present two novel fungal species, *Dictyosporium thecatum* and *Melomastia shenzhenensis* and three new records of *Melomastia* species. All the taxonomic novelties were confirmed through molecular phylogenetic analyses and morphological evidence.

## ﻿Materials and methods

### ﻿Sample collection, examination and isolation of fungi

A fungal collection from dead plant materials were conducted from different sampling sites in Shenzhen Bay, Guangdong Province, China during January to June 2025. Dead twigs, branches were collected and those samples were taken into the laboratory in polythene bags and paper envelopes for examination.

Micro-morphological characteristics including structure and size of ascomata, conidiomata, asci, ascospores and conidia were observed using an OLYMPUS SZ61 compound microscope. Images of these fungal micro-structures were acquired with a Canon EOS 600D digital camera equipped with a Nikon ECLIPSE 80i compound microscope. All the measurements were taken using NIS-Elements version 5.10 imaging software when capturing photographs. Photographs were processed and photographic plates were assembled using Adobe Photoshop 2021 (Adobe Systems, San Jose, CA).

Pure fungal cultures were obtained through single spore isolation following Senanayake et al. (2020). Single germinating spores were transferred on to fresh PDA (Potato Dextrose Agar) plates and incubated in the dark at 25 °C. Herbarium specimens were deposited in the Herbarium of Shenzhen University (**SZU**), China. The living cultures were deposited in the Microbial Culture Collection at Shenzhen University (**MBSZU**), China. Facesoffungi numbers are provided as explained in Jayasiri et al. (2015) and Index Fungorum identifiers were obtained by registering with Index Fungorum (2025).

### ﻿DNA extraction, PCR amplification and sequencing

Initially, pure fungal cultures were grown in PDA and kept the dark at temperatures ranging from 25 to 27 °C. Fresh fungal mycelia were scraped from these fungal cultures under axenic conditions. Subsequently, fungal genomic DNA was extracted from mycelia using Biospin fungus genomic DNA kit (BioFlux, P.R. China) according to the manufacturer’s instructions. DNA was kept at 4 °C for the DNA amplification of genes and maintained at –20 °C for long-term storage.

DNA amplification was performed by polymerase chain reaction (PCR). Both forward and reverse primers of four loci that used for the PCR amplification, namely, internal transcribed spacers (ITS), large subunit rDNA (LSU), small subunit rDNA (SSU) and translation elongation factor 1-α (*tef*1-α) regions were listed in Table 1. The PCR thermal cycle programmes for LSU, SSU, ITS and *tef1* amplification were followed as in Wu et al. (2024). The final reaction volume of the PCR mixture was 25 µl containing 1 µl of DNA template, 1 µl each of the forward and reverse primer, 9.5 µl of double-distilled water (ddH_2_O) and 12.5 µl of 2× taq PCR Master Mix (mixture of DNA Polymerase, dNTPs, Mg2+ and optimised buffer; CoWin Biosciences, Jiangsu, China). The PCR product quality was tested on 1% agarose electrophoresis gels stained with ethidium bromide. PCR purification and sequencing of amplified PCR products were carried out at Beijing Liuhe BGI Genomics Co., Ltd., China.

**Table 1.** Forward and reverse primers information of ITS, LSU, SSU and *tef*1-α regions.

Locus	Primers	Reference
**ITS**	**Forward: ITS5** TCCTCCGCTTATTGATATGC	White et al. (1990)
	**Reverse: ITS4** GGAAGTAAAAGTCGTAACAAGG	
**LSU**	**Forward: LR0R** GTACCCGCTGAACTTAAGC	Vilgalys and Hester (1990)
	**Reverse: LR5** ATCCTGAGGGAAACTTC	
**SSU**	**Forward: NS1** GTAGTCATATGCTTGTCTC	White et al. (1990)
	**Reverse: NS4** CTTCCGTCAATTCCTTTAAG	
***tef1-α***	**Forward: EF1-983F** GCYCCYGGHCAYCGTGAYTTYAT	Rehner and Buckley (2005)
	**Reverse: EF1-2218R** ATGACACCRACRGCRACRGTYTG	

### ﻿Phylogenetic analyses

Initially, resulted DNA sequence data was matched with available sequence data in the GenBank, based on the BLAST (http://www.ncbi.nlm.nih.gov/) search tool. Representative fungal taxa that are closely related to our new strains were retrieved from GenBank and recent publications (Li et al. 2022; Kularathnage et al. 2023; Tennakoon et al. 2023; Shu et al. 2024; Hongsanan et al. 2025; Wang et al. 2025). Individual gene sequence data were aligned with MAFFT v. 7 (Katoh et al. 2019) and manually improved where necessary using BioEdit v. 7.0.5.2 (Hall 1999). Newly-generated sequence data were deposited in GenBank. The accession numbers used in the phylogenetic analyses were mentioned in Tables 2, 3.

**Table 2.** Taxa names, strain numbers and corresponding GenBank accession numbers of the taxa included in the phylogenetic analysis of *Melomastia*.

Taxa name	Culture accession number	GenBank accession numbers			
		LSU	SSU	ITS	*tef1-α*
*Acrospermum adeanum*	M 133	EU940104	EU940031	EU940180	–
*Acrospermum compressum*	M 151	EU940084	EU940012	EU940161	–
*Acrospermum graminum*	M 152	EU940085	EU940013	EU940162	–
*Anisomeridium phaeospermum*	MPN539	JN887394	JN887374	–	JN887418
*Anisomeridium ubianum*	MPN94	–	JN887379	–	JN887421
*Dyfrolomyces chromolaenae*	MFLUCC 17-1434^T^	–	MT214413	–	MT235800
*Dyfrolomyces tiomanensis*	MFLUCC 13-0440 ^T^	KC692156	KC692155	–	KC692157
*Melomastia aquilariae*	ZHKUCC 23-0073 ^T^	OR807856	OR807854	–	OR832867
*Melomastia beihaiensis*	KUMCC 21-0084 ^T^	MZ726990	MZ727002	MZ726997	OK043822
***Melomastia beihaiensis***	**MBSZU 25-048**	**PX271110**	**PX271105**	**PX308635**	**PX273902**
*Melomastia clematidis*	MFLUCC 17-2092 ^T^	MT214607	MT226718	MT310651	MT394663
*Melomastia diqingensis*	KUMCC 21-0536	OQ170873	OQ168224	OQ158951	OR613413
*Melomastia distoseptata*	NFCCI 4377	MH971236	–	MK024391	–
*Melomastia fulvicomae*	MFLUCC 17-2083 ^T^	MT214608	MT226719	MT310652	–
*Melomastia fusispora*	CGMCC 3.20618 ^T^	OK623464	OK623494	OK623480	OL335189
*Melomastia fusispora*	UESTCC 21.0001 ^T^	OK623465	OK623495	OK623481	OL335190
***Melomastia fusispora***	**MBSZU 25-050**	**PX271112**	**PX271106**	**PX289102**	**PX273903**
*Melomastia guangdongensis*	GMBCC1046 ^T^	PQ530970	PQ530975	–	PQ559185
*Melomastia hydei*	MBSZU 25-003 ^T^	PQ844642	PQ844644	PQ849544	PQ868900
*Melomastia hydei*	MBSZU 25-004	PQ844643	PQ844645	PQ849545	PQ868901
***Melomastia hydei***	**MBSZU 25-049**	**PX271111**	–	**PX289101**	–
*Melomastia italica*	MFLUCC 15-0160 ^T^	MG029458	MG029459	–	–
*Melomastia loropetalicola*	ZHKUCC 22-0174 ^T^	OQ379412	OQ379411	–	–
*Melomastia maolanensis*	GZCC 16-0102 ^T^	KY111905	KY111906	–	KY814762
*Melomastia maomingensis*	ZHKUCC 23-0038 ^T^	PP809724	PP809704	OR825372	PP812255
*Melomastia maomingensis*	ZHKUCC 23-0055	PP809725	PP809705		PP812256
*Melomastia neothailandica*	MFLU 17-2589 ^T^	NG068294	–	–	–
*Melomastia oleae*	CGMCC 3.20619 ^T^	OK623466	OK623496	OK623482	OL335191
*Melomastia oleae*	UESTCC 21.0003	OK623467	OK623497	–	OL335192
*Melomastia phetchaburiensis*	MFLUCC 15-0951 ^T^	MF615402	MF615403	–	–
*Melomastia puerensis*	ZHKUCC 23-0803	OR922310	OR922341	OR941078	OR966285
*Melomastia puerensis*	ZHKUCC 23-0802 ^T^	OR922309	OR922340	OR941077	OR966284
*Melomastia pyriformis*	ZHKUCC 22-0175 ^T^	OP791870	OP739334	–	OQ718392
*Melomastia rhizophorae*	JK 5439 A	GU479799	GU479766	–	GU479860
*Melomastia septata*	MFLUCC 22-0112 ^T^	OP749870	–	–	OP760198
***Melomastia shenzhenensis***	**MBSZU 25-051^T^**	**PX271109**	**PX271104**	**PX308634**	**PX273901**
*Melomastia sichuanensis*	CGMCC 3.20620^T^	OK623469	OK623500	–	OL335195
*Melomastia sinensis*	MFLUCC 17-1344 ^T^	MG836699	MG836700	–	–
*Melomastia thailandica*	MFLUCC 15-0945 ^T^	KX611366	KX611367	–	–
*Melomastia thamplaensis*	MFLUCC 15-0635 ^T^	KX925435	KX925436	–	KY814763
*Melomastia winteri*	CGMCC 3.20621	OK623471	OK623502	OK623485	OL335197
*Melomastia yunnanensis*	GMBCC1009T	PQ530973	PQ530978	–	PQ559188
*Muyocopron castanopsis*	MFLUCC 14-1108	KU726965	KU726968	–	MT136753
*Muyocopron dipterocarpi*	MFLU 17-2608	KU726966	KU726969	NR_168252	MT136754
*Muyocopron heveae*	MFLUCC 17-0066 ^T^	MH986832	MH986828	MH986836	–
*Muyocopron lithocarpi*	MFLUCC 14-1106 ^T^	KU726967	KU726970	NR_168253	MT136755
*Palawania thailandensis*	MFLICC 14-1121 ^T^	KY086493	KY086495	MT137787	–
*Palawania thailandensis*	MFLU 16-1873	KY086494	–	MT137788	–
*Stigmatodiscus enigmaticus*	CBS 132036 ^T^	KU234108	KU234130	KU234108	MH756078
*Stigmatodiscus labiatus*	CBS 144700/AP 6516 ^T^	MH756065	MH756065	MH756065	MH756083
*Stigmatodiscus oculatus*	CBS 144701	MH756069	–	MH756069	MH756086
*Stigmatodiscus pruni*	CBS 142598 ^T^	KX611110	KX611110	KX611110	KX611111

Remarks: The newly-generated sequences are indicated in red bold, ex-types indicated in superscript T and “-” indicated unavailable data.

**Table 3.** Taxa names, strain numbers and corresponding GenBank accession numbers of the taxa included in the phylogenetic analysis of *Dictyosporium*.

Taxa name	Culture accession number	GenBank accession numbers		
		ITS	LSU	*tef1-α*
*Dictyosporium alatum*	ATCC 34953 ^T^	NR_077171	DQ018101	–
*Dictyosporium aquaticum*	CBS 21460 ^T^	KM610236	–	–
*Dictyosporium bulbosum*	yone 221 ^T^	LC014544	AB807511	AB808487
*Dictyosporium digitatum*	KUMCC 17-0269 ^T^	MH388344	MH376716	MH388378
*Dictyosporium duliujiangense*	GZCC 19-0426 ^T^	OQ842725	MW133815	OQ850746
*Dictyosporium elegans*	NBRC 32502 ^T^	DQ018087	DQ018100	–
*Dictyosporium guangdongense*	ZHKUCC 24-0002 ^T^	PP326190	PP326213	–
*Dictyosporium guttulatum*	MFLUCC 16-0258 ^T^	MH388345	MH376717	MH388379
*Dictyosporium hongkongensis*	KUMCC 17-0268 ^T^	MH388346	MH376718	MH388380
*Dictyosporium hughesii*	KT 1847 ^T^	LC014548	AB807517	AB808493
*Dictyosporium karsti*	MFLU 18-2282	OR225025	OP099521	OR140390
*Dictyosporium krabiense*	MFLU 16-1890T	–	MH376719	MH388381
*Dictyosporium meiosporum*	MFLUCC 10-0131 ^T^	KP710944	KP710945	–
*Dictyosporium muriformis*	GZCC 20-0006 ^T^	MT002304	MN897834	MT023011
*Dictyosporium nigroapice*	BCC 3555	DQ018085	–	–
*Dictyosporium olivaceosporum*	KH 375 ^T^	LC014542	AB807514	AB808490
*Dictyosporium palmae*	CBS-H 22129	–	KX555648	–
*Dictyosporium pandanicola*	MFLU 16-1886 ^T^	MH388347	MH376720	MH388382
*Dictyosporium* sp.	MFLUCC 15-0629	MH381766	MH381775	MH388819
*Dictyosporium sexualis*	MFLUCC 10-0127 ^T^	KU179105	KU179106	–
*Dictyosporium stellatum*	CCFC 241241 ^T^	NR_154608	JF951177	–
*Dictyosporium strelitziae*	CBS 123359 ^T^	NR_156216	FJ839653	–
*Dictyosporium tetrasporum*	KT 2865	LC014551	AB807519	AB808495
*Dictyosporium thailandicum*	MFLUCC 13-0773 ^T^	KP716706	KP716707	–
***Dictyosporium thecatum***	**MBSZU 25-047 ^T^**	**PX289103**	**PX271113**	**PX273904**
*Dictyosporium tratense*	MFLUCC 17-2052 ^T^	MH381767	MH381776	MH388820
*Dictyosporium tubulatum*	MFLUCC 15-0631 ^T^	MH381769	MH381778	MH388822
*Dictyosporium variabilisporum*	ZHKUCC 24-0003	PP326192	PP326215	PP333112
*Dictyosporium wuyiense*	CGMCC 3.18703 ^T^	KY072977	–	–
*Dictyosporium zhejiangense*	MW-2009a ^T^	FJ456893	–	–
*Immotthia bambusae*	KUN-HKAS 112012AI ^T^	MW489455	MW489450	MW504646
*Immotthia bambusae*	KUN-HKAS 112012AII	MW489456	MW489451	MW504647
*Pseudodictyosporium elegans*	CBS 688.93 ^T^	DQ018099	DQ018106	–
*Pseudodictyosporium thailandica*	MFLUCC 16-0029 ^T^	KX259520	KX259522	KX259526
*Pseudodictyosporium wauense*	NBRC 30078 ^T^	DQ018098	DQ018105	–
*Pseudodictyosporium wauense*	DLUCC 0801	MF948622	MF948630	MF953165

Remarks: The newly-generated sequences are indicated in red bold, ex-types indicated in superscript T and “-” indicated unavailable data.

Evolutionary models for phylogenetic analyses were selected for each locus using MrModelTest v. 3.7 (Posada and Crandall 1998) under the Akaike Information Criterion (AIC). Maximum-Likelihood (ML) analysis was carried out in online portal CIPRES Science Gateway v.3.3 (http://www.phylo.org/portal2/; Miller et al. (2010)) using RAxML-HPC2 on XSEDE (8.2.12) tool (Stamatakis 2014). The default settings were adapted, except for the selection of GAMMA nucleotide substitution model and 1,000 rapid bootstrap replicates. Bayesian analysis was conducted with MrBayes v. 3.1.2 (Huelsenbeck and Ronqvist 2001). Parameters for Bayesian Inference: Markov chains were set to run 1,000,000 generations and resulting trees were sampled every 100^th^ generation (printfreq = 100) and 10,000 trees were obtained. Initial trees were discarded (20% burn-in value) and the remaining trees were used to evaluate posterior probabilities (PP) in the majority rule consensus tree. Phylograms were visualised in FigTree v.1.4.0 (http://tree.bio.ed.ac.uk/software/figtree/; Rambaut (2014)) and the tree was edited using Microsoft PowerPoint (2010).

In addition, pairwise similarity identity values were calculated for *Dictyosporium* and *Melomastia* genera. Sequence Demarcation Tool version 1.2 (SDT v.1.2) (Muhire et al. 2014) was used to generate pairwise nucleotide sequence identity matrix. Parameters of Pairwise identity calculation were set as ‘ClustalW’ for alignment programmes without specifying ‘cluster sequences using a neighbour-joining tree’ option. Sequence identity scores were saved as a matrix and delimited characters, such as commas or tabs, separated in each field. Finally, we visualised the pairwise similarity identity matrix as plots (Figs 2a, b, 4a, b) and included a colour key representing the correspondence between pairwise identity values and the colours displayed in the matrix.

## ﻿Results

### ﻿Phylogenetic analyses

**1. Phylogenetic analyses: *Dictyosporium***

**Figure 1.**
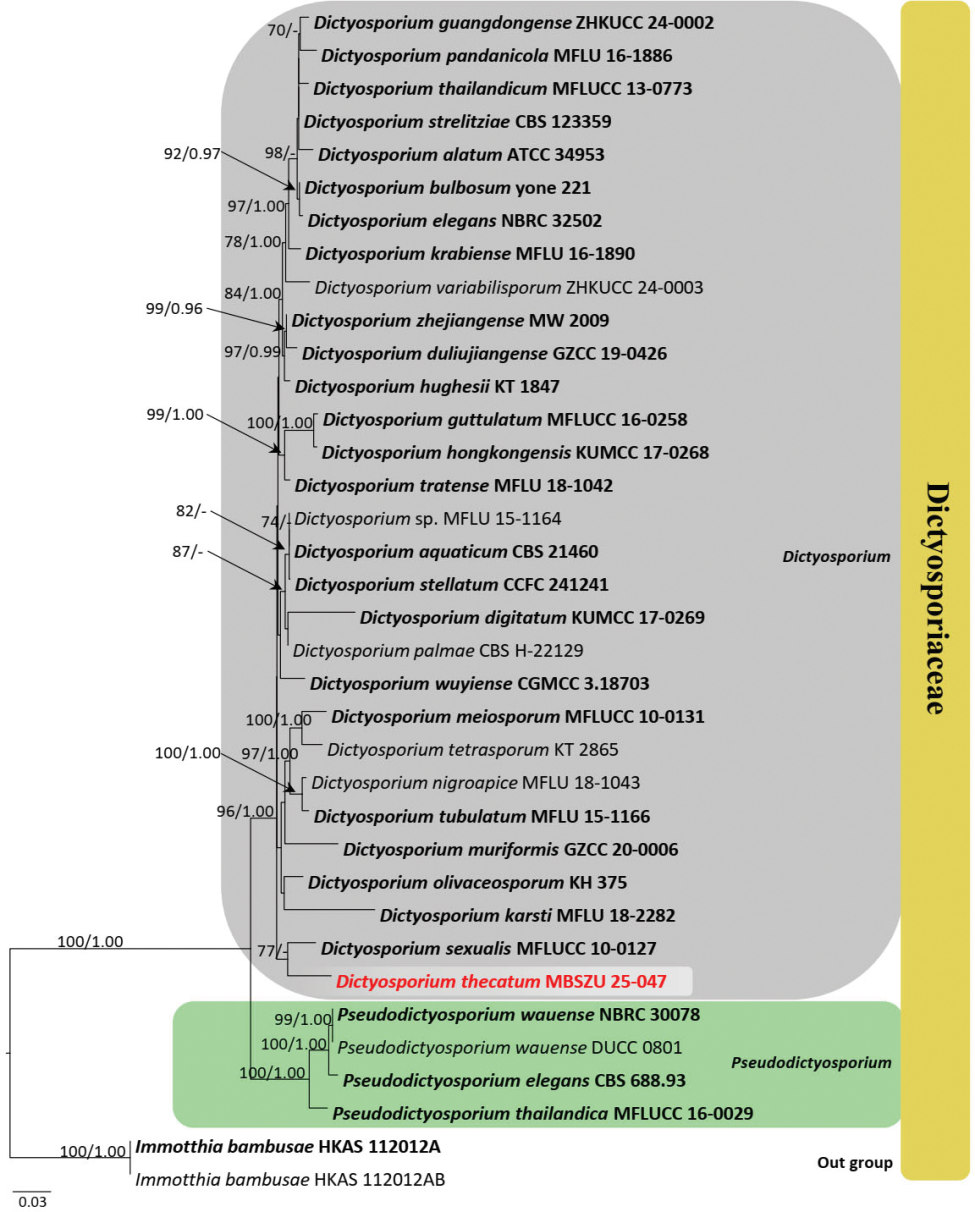
The phylogram, generated from Maximum Likelihood analysis, is based on combined ITS, LSU and *tef1-α* sequence data. The tree is rooted with *Immotthia bambusae* (HKAS 112012A and HKAS 112012B). The new strains are indicated in red and ex-type strains are in bold. Bootstrap support values ≥ 75% from the Maximum Likelihood (ML) and Bayesian Posterior Probabilities (BYPP) values ≥ 0.95 are given above the nodes, respectively.

Phylogenetic relationships of a new strain were assessed, based on concatenated ITS, LSU and *tef*1-α gene regions of 36 strains in Dictyosporiaceae. The final alignment consisted of combined ITS (1–610 bp), LSU (611–1895 bp), and *tef*1-α (1896–2820 bp) sequence data, including gaps. Two strains of *Immotthia bambusae* (HKAS 112012A and HKAS 112012B) served as outgroup taxa. The best scoring RAxML tree is shown in Fig. 1. The RAxML analysis of the combined dataset yielded a best-scoring tree (Fig. 1). The final ML optimisation likelihood value was -10451.848250. There were 32.80% undetermined characters or gaps and 729 distinct alignment patterns. The estimated base frequencies were A = 0.238697, C = 0.250582, G = 0.269387 and T = 0.241335; the substitution rates were AC = 1.242166, AG = 3.644387, AT = 2.048736, CG = 1.165862, CT = 9.067381 and GT = 1.000; the proportion of invariable sites I = 0.526947; and the gamma distribution shape parameter was *a* = 0.675504. The Bayesian analysis resulted in 10,000 trees after 1,000,000 generations.

**Figure 2.**
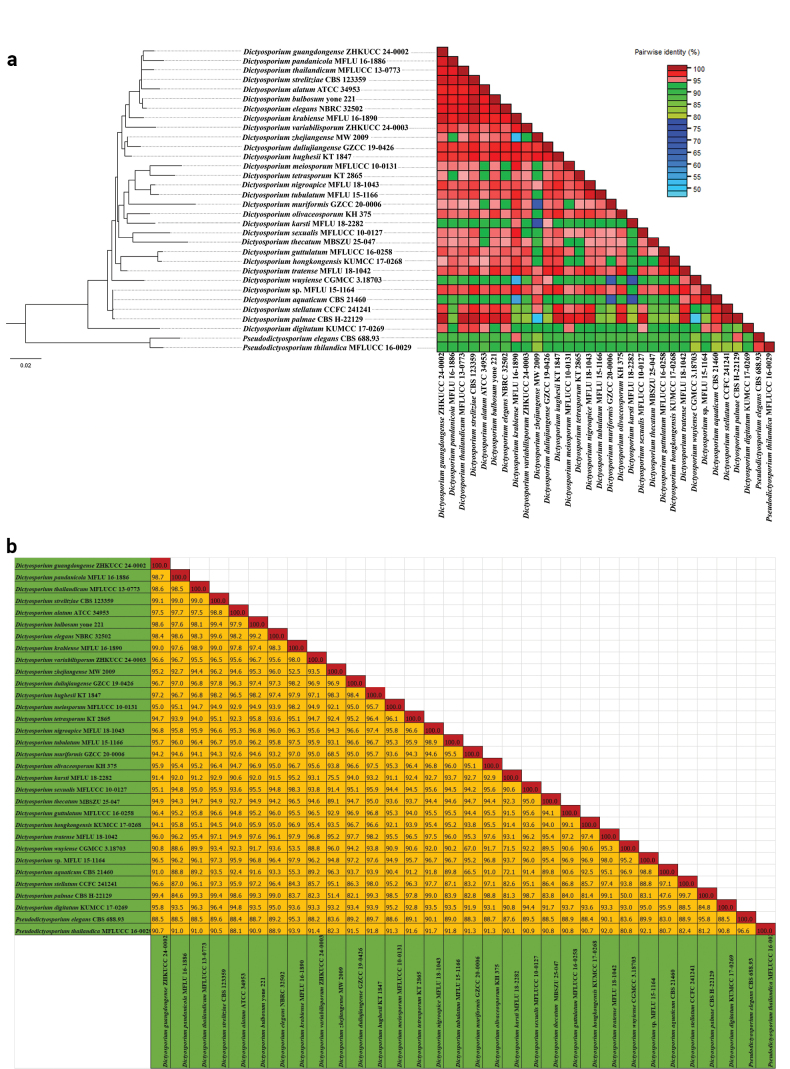
a. Colour-coded pairwise identity matrix, based on combined ITS, LSU, and *tef1-α* from 30 strains of *Dictyosporium* and two strains of *Pseudodictyosporium* species. Each coloured cell represents a percentage identity score between two sequences (one indicated horizontally to the left and the other vertically at the bottom). A coloured key indicates the correspondence between pairwise identities and the colours displayed in the matrix; b. Pairwise identity score distribution plot, based on combined ITS, LSU, and *tef1-a* from 30 strains of *Dictyosporium* and two strains of *Pseudodictyosporium* species.

Maximum Likelihood and Bayesian analyses generated similar tree topology and concurred with previous studies (Tennakoon et al. 2023; Shu et al. 2024; Wang et al. 2025). Thirty strains of *Dictyosporium* species formed a monophyletic clade and nested within Dictyosporiaceae. In the current phylogram, a new strain, MBSZU 25-047, clusters within *Dictyosporium* species. Further, MBSZU 25-047 separates from other *Dictyosporium* species and forms a distinct basal clade with *D. sexualis* (MFLUCC 10-0127) with 77% ML and 0.75 BYPP statistical support (Fig. 1). The pairwise similarity values were calculated, based on concatenated ITS, LSU and *tef*1-α gene regions of 30 strains of *Dictyosporium* and two strains of *Pseudodictyosporium* species. According to the pairwise similarity plot, all taxa except for *D. sexualis* (MFLUCC 10-0127) and *D. krabiense* (MFLU 16-1890) show less than 95% pairwise identity with the new strain (MBSZU 25-047), which is represented by a pink-to-green gradient in the colour-coded matrix (Fig. 2a, b).

### ﻿2. Phylogenetic analyses: *Melomastia*

**Figure 3.**
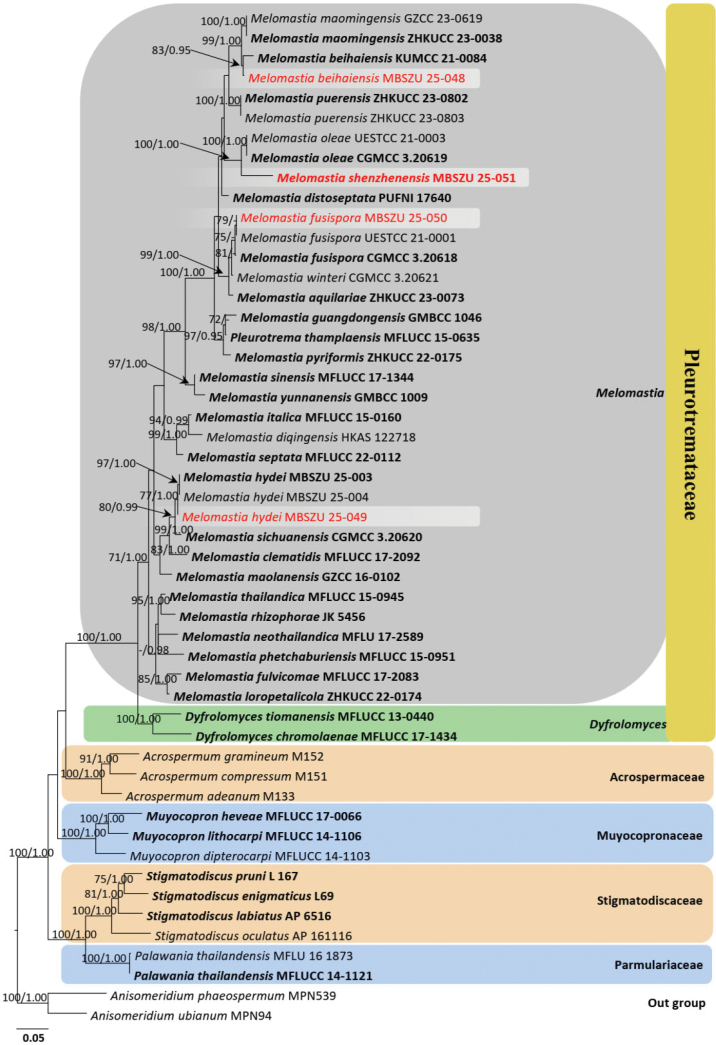
The phylogram generated from Maximum Likelihood analysis is based on combined LSU, SSU, ITS and *tef1-α* sequence data. The tree is rooted with *Anisomeridium phaeospermum* (MPN539) and *A. ubianum* (MPN94). The new strains are indicated in red and ex-type strains are in bold. Bootstrap support values ≥ 70% from the Maximum Likelihood (ML) and Bayesian Posterior Probabilities (BYPP) values ≥ 0.95 are given above the nodes, respectively.

Phylogenetic relationships of four new strains were assessed, based on concatenated LSU, SSU, ITS and *tef*1-α gene regions of 51 strains in Pleurotremataceae, Acrospermaceae and Muyocopronaceae. The analysed alignment consisted of combined LSU (1–990 bp), SSU (991–2805 bp), ITS (2806–3740 bp) and *tef*1-α (3741–4995 bp) sequence data, including gaps. *Anisomeridium phaeospermum* (MPN539) and *A. ubianum* (MPN94) served as outgroup taxa. The best scoring RAxML tree is shown in Fig. 3. The RAxML analysis of the combined dataset yielded a best-scoring tree (Fig. 3). The final ML optimisation likelihood value was -27277.751366. There were 48% undetermined characters or gaps and 2089 distinct alignment patterns. The estimated base frequencies were A = 0.234511, C = 0.266661, G = 0.291035 and T = 0.207793; the substitution rates were AC = 1.097961, AG = 2.029065, AT = 1.343076, CG = 1.101087, CT = 5.107863 and GT = 1.000; the proportion of invariable sites I = 0.439505; and the gamma distribution shape parameter was *a* = 0.945325. The Bayesian analysis resulted in 20,000 trees after 2,000,000 generations.

**Figure 4.**
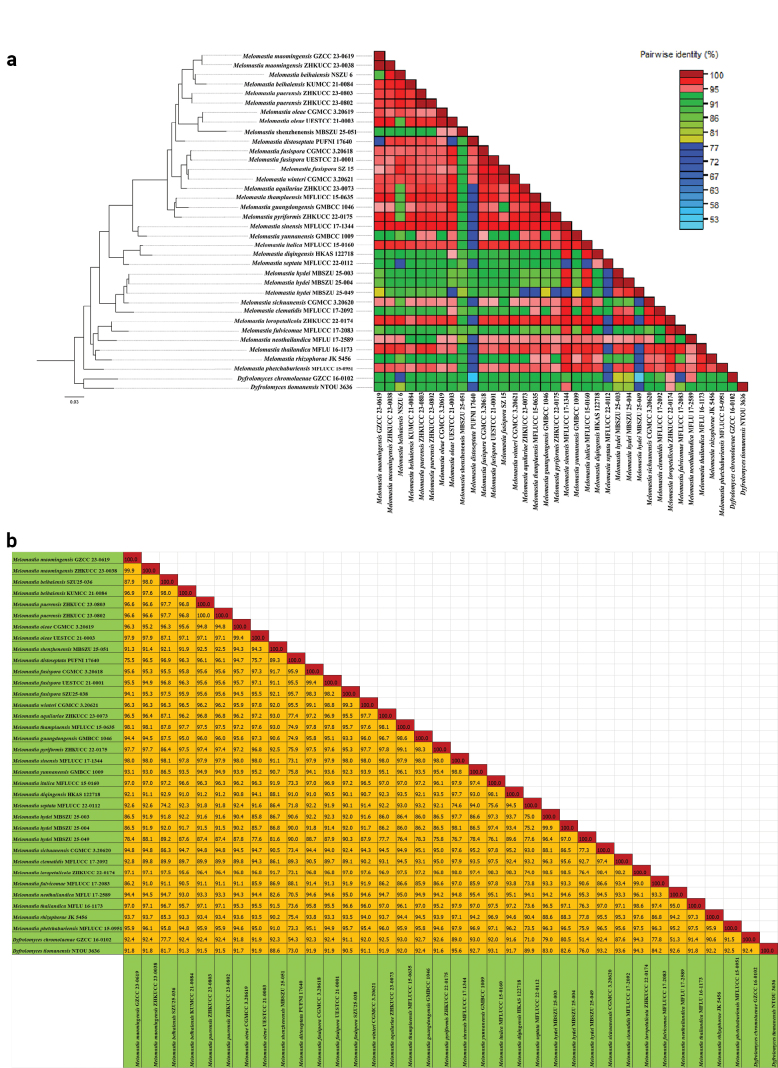
a. Colour-coded pairwise identity matrix, based on combined LSU, SSU, ITS and *tef1-α* from 34 strains of *Melomastia* and two *Dyfrolomyces* species. Each coloured cell represents a percentage identity score between two sequences (one indicated horizontally to the left and the other vertically at the bottom). A coloured key indicates the correspondence between pairwise identities and the colours displayed in the matrix; b. Pairwise identity score distribution plot, based on combined LSU, SSU, ITS and *tef1-α* from 34 strains of *Melomastia* and two *Dyfrolomyces* species.

Maximum Likelihood and Bayesian analyses generated similar findings and concurred with previous studies (Li et al. 2022; Kularathnage et al. 2023; Du et al. 2024; Hongsanan et al. 2025). *Dyfrolomyces* and *Melomastia* species formed two distinct clades and nested within Pleurotremataceae in our phylogeny and previous studies (Kularathnage et al. 2023; Du et al. 2024; Xu et al. 2024). The new strain, MBSZU 25-051, clusters within *Melomastia* and forms an independent lineage sister to *M. oleae* with strong support (100% ML and 1.00 BYPP). Further, our strain, MBSZU 25-048 shows close phylogenetic relationships with the type strains of *M. beihaiensis* (KUMCC 21-0084) and *M. maomingensis* (ZHKUCC 23-0038 and ZHKUCC 23-0055). Another new strain, MBSZU 25-049 clusters closely related to *M. hydei* (MBSZU 25-003 and MBSZU 25-004) with 77% ML and 1.00 BYPP statistical support. In addition, MBSZU 25-050 clusters within a group that includes *M. aquilariae*, *M. fusispora*, *M. winteri*. However, the new strain, MBSZU 25-050, is closely related to *M. fusispora* (CGMCC 3.20618 and UESTCC 21.0001) with 79% ML and 0.66 BYPP statistical support. The pairwise values were calculated, based on concatenated LSU, SSU, ITS and *tef*1-α gene regions of 34 strains of *Melomastia* and two strains of *Dyfrolomyces* species. The new strain (MBSZU 25-051) shows the highest pairwise identity (94.3%, indicated in pink) with two *M. oleae* strains: CGMCC 3.20619 (ex-type) and UESTCC 21.0003 (Fig. 4a, b). Further details of the pairwise identity scores of new host records are mentioned in ‘Notes’ section of each taxon.

### ﻿Taxonomy

**Dothideomycetes O.E. Erikss. & Winka**

**Pleosporales Luttr. ex M.E. Barr**

**Dictyosporiaceae Boonmee & K.D. Hyde**

Dictyosporiaceae was formally introduced by Boonmee et al. (2016) with *Dictyosporium* Corda as the type genus and *D. elegans* Corda as the type species. The family had been initially proposed by Liu et al. (2015) as “Dictyosporaceae”, but it was not validly published. The family comprises holomorphic group of species including hyphomycetous asexual morph and sexual morph with perithecial ascomata (Boonmee et al. 2016). Species of this family are saprobic on plant debris, dead or rotting wood in aquatic and terrestrial environments across temperate, tropical and subtropical zones worldwide (Boonmee et al. 2016; Yang et al. 2018; Liu et al. 2023; Tennakoon et al. 2023; Farr and Rossman 2025). Twenty-three genera are accepted in Dictyosporiaceae, the majority of which exhibit as hyphomycetous asexual morphs (Wang et al. 2025).

### ﻿*Dictyosporium* Corda

*Dictyosporium* was established by Corda (1836) with the type *D. elegans*. Species of this genus are identified from wood, decaying leaves and other plant matter in terrestrial and freshwater habitats worldwide (Boonmee et al. 2016; Tennakoon et al. 2023; Wang et al. 2025). The asexual morph is characterised by sporodochial colonies, micronematous to macronematous, branched conidiophores, sometimes reduced to conidiogenous cells, cheiroid, digitate, complanate, brown conidia with several parallel rows of cells with or without appendages. The sexual morph is characterised by globose to subglobose ascomata, bitunicate, cylindrical asci and hyaline, fusiform, uniseptate ascospores with or without a sheath (Goh et al. 1999; Boonmee et al. 2016; Yang et al. 2018; Liu et al. 2023; Tennakoon et al. 2023; Wang et al. 2025). Ninety-one species are listed in the Index Fungorum (2025).

#### ﻿ Dictyosporium thecatum

Taxon classificationFungiAscomycotaDothideomycetes

N.I. de Silva, Tennakoon & S. Hongsanan sp. nov.

BB466463-BBD4-594B-BA39-E828FD274328

Index Fungorum: IF904326

Facesoffungi Number: FoF07238

Figs 5, 6

##### Etymology.

The name refers to the ascospores having a clear sheath and the Latin word “thecatum” means “possessing a sheath”.

**Figure 5.**
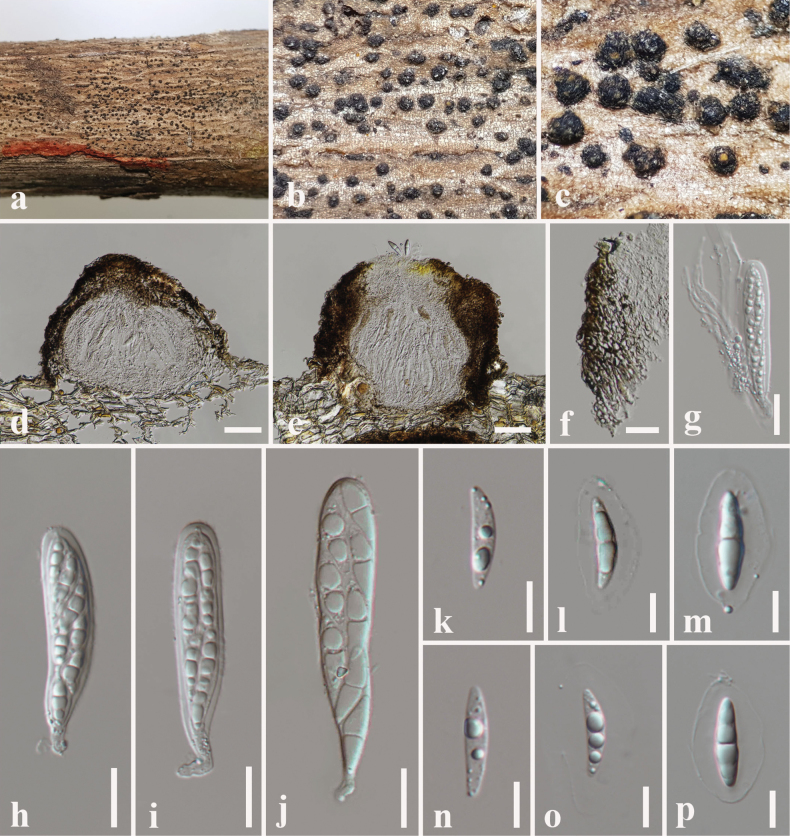
*Dictyosporium thecatum* (SZU 25-035, holotype). a. The plant substrate; b, c. Appearance of ascomata on substrate; d, e. Vertical sections through ascomata; f. Peridium; g. Pseudoparaphyses with young ascus; h–j. Asci; k–p. Ascospores. Scale bars: 50 μm (d, e); 20 μm (f–j); 10 μm (k–p).

##### Holotype.

SZU 25-035

**Figure 6.**
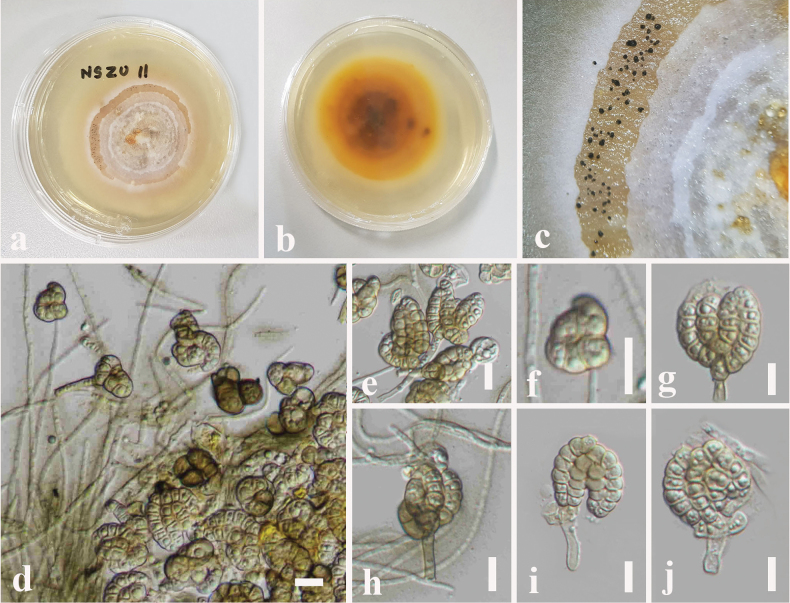
*Dictyosporium thecatum* (MBSZU 25-047, ex-type). a, b. Colonies on PDA (front and reverse view); c. Sporodochia on PDA; d. Different stages of developing conidia with mycelia; e–j. Conidiophores with conidia. Scale bars: 10 μm (d–j).

##### Description.

***Saprobic*** on dead branch of *Volkameria inermis* L. **Sexual morph: *Ascomata*** 160–220 μm high × 190–240 µm diam. (x̄ = 180 × 210 μm, n = 10), dark brown to black, uniloculate, superficial, scattered to gregarious, globose to subglobose with an apical ostiole. ***Peridium*** 30–55 µm wide, (x̄ = 42 μm, n = 15), composed of dark brown cells of *textura angularis* outer layer, pale brown to hyaline cells of *textura angularis* inner layer. ***Hamathecium*** 1–2 µm wide, hyaline, cellular, filamentous, branched, septate pseudoparaphyses. ***Asci*** 77–98 × 14–17 µm (x̄ = 85 × 15 μm, n = 20), 8-spored, bitunicate, fissitunicate, cylindrical, elongate, apically rounded with an ocular chamber with short pedicelled. ***Ascospores*** 22–25 × 4–7 µm (x̄ = 23.5 × 5.6 μm, n = 30), hyaline, overlapping, uni-seriate, fusiform, slightly curved, 1-septate, constricted at the septum, guttulate, smooth-walled, surrounded by a 5–7 μm thick mucilaginous sheath. **Asexual morph**: Hyphomycetous. ***Colonies*** on PDA: sporodochial, effuse or compact, brown or black, immersed mycelia. ***Conidiophores*** 10–18 × 2–4 µm (x̄ = 13 × 3 μm, n = 5), hyaline, pale brown, micronematous, mononematous, subcylindrical, flexuous, smooth, mix with vegetative hyphae. ***Conidiogenous cells*** 8–10 × 6–8 µm (x̄ = 9 × 7 μm, n = 5), holoblastic, monoblastic, integrated, determinate, terminal. ***Conidia*** 17–25 × 13–18 µm (x̄ = 19 × 15 μm, n = 30), complanate, cheiroid, consisting of 10–28 cells arranged in 3–5 tightly appressed rows, 2–8 euseptate in each column with a cuneiform or swollen basal cell, acrogenous, guttulate, smooth-walled.

##### Culture characteristics.

Colonies on PDA reaching 28 mm diameter after 1 week at 25 °C, colonies from above: circular, margin undulate, dense, flat, white at the margin, pale brown layer with black, compact sporodochia, whitish-cream in the centre with fluffy, aerial mycelia; reverse: cream at the margin, brown in the centre. Sporulated on PDA after 2 weeks at 25 °C.

##### Material examined.

China • Guangdong Province, Shenzhen, on dead branch of *Volkameria inermis* L. (Lamiaceae), 11 June 2025, N. I de Silva NSZU11 (SZU 25-035, **holotype**), ex-type living culture, MBSZU 25-047.

##### Notes.

*Dictyosporium thecatum* (MBSZU 25-047) forms a distinct basal group comprising ex-type strain of *D. sexualis* (MFLUCC 10-0127) with 77% ML, 0.75 BYPP statistical support (Fig. 1). *Dictyosporium thecatum* differs from *D. sexualis* in having smaller ascomata (160–220 high μm × 190–240 µm diam. vs. 235–270 μm high × 250–285 μm diam.), asci (77–98 × 14–17 µm vs. 100–145 × 10– 14 μm) and ascospores (22–25 × 4–7 µm vs. 36–48 × 6–8 μm) (Boonmee et al. 2016). Further, *Dictyosporium thecatum* has a larger mucilaginous sheath surrounding ascospores (5–7 μm) than *D. sexualis* (2–4.5(–5) μm) (Boonmee et al. 2016). However, no asexual morph has been reported for *D. sexualis*. The comparison of nucleotide substitutions between *D. thecatum* and the ex-type *D. sexualis* (MFLUCC 10-0127) showed 9.46% (53/560) differences (without gaps) in the ITS gene region. However, no *tef1-α* sequence data of *D. sexualis* (MFLUCC 10-0127) are available in GenBank. Based on the colour-coded pairwise identity matrix, *Dictyosporium thecatum* (MBSZU 25-047) shares 95% identity (light pink) with *Dictyosporium sexualis* (MFLUCC 10-0127) (Fig. 2a, b). *Dictyosporium sexualis* was introduced from dead branches of an unidentified plant in Doi Tung, Chiang Rai, Thailand (Boonmee et al. 2016). We, therefore, introduce *Dictyosporium thecatum* as a new species, based on morphology, phylogeny and host-geographical associations.

### ﻿Dothideomycetes orders *incertae sedis*

**Dyfrolomycetales K.L. Pang et al.**

**Pleurotremataceae Walt. Watson (= Dyfrolomycetaceae K.D. Hyde et al.)**

Watson (1929) established Pleurotremataceae to include *Pleurotrema* with the type *P. polysemum*. Another familial rank, Dyfrolomycetaceae which was introduced by Pang et al. (2013) to accommodate *Dyfrolomyces* species was later synonymised under Pleurotremataceae (Maharachchikumbura et al. 2016). Pleurotremataceae was previously assigned in to Xylariales, Sordariomycetes (Barr 1994) and later transferred to Dothideomycetes after re-examination of herbarium materials (Maharachchikumbura et al. 2016). Three genera are accepted in in Pleurotremataceae, i.e. *Dyfrolomyces*, *Melomastia* and *Pleurotrema* (Hongsanan et al. 2020; Wijayawardene et al. 2022). The sexual morph is characterised by immersed ascomata, with a clypeus on the substrate, cylindrical asci and multi-septate ascospores with or without a sheath (Watson 1929; Barr 1994).

### ﻿*Melomastia* Nitschke & Sacc.

*Melomastia* was introduced by Saccardo (1875) to accommodate *M. mastoidea* (= *Melomastia friesii*). Species Fungorum lists 66 epithets for *Melomastia* (accessed on 1 August 2025), yet most remain without molecular data. The sexual morph is characterised by semi-immersed, globose to subglobose, black, ostiolate ascomata with erumpent apex, cylindrical asci and hyaline, ovoid or cylindrical, 1–10-septate, ascospores with or without a gelatinous sheath (Kang et al. 1999; Hongsanan et al. 2020; de Silva et al. 2022; Li et al. 2022; Kularathnage et al. 2023; Hongsanan et al. 2025). Since the high degree of morphological overlap of ascospore characters between *Melomastia* and *Dyfrolomyces*, delimitation of two genera became challenging. Thus, based on the morphology and multigene phylogeny, 11 *Dyfrolomyces* species transferred to *Melomastia* (Li et al. 2022). However, *M. tiomanensis* and *M. chromolaenae* transferred to *Dyfrolomyces*, based on the most recent phylogenetic evidence by Kularathnage et al. (2023) as they form a well-supported basal clade separated from the remainder of *Melomastia* species. Further, this was supported by the presence of spindle-shaped ascospores with 6–11-septa and acute, tapering ends, that has not been observed in other *Melomastia* species (Kularathnage et al. 2023). The current phylogenetic analyses represented a topology consistent with recent studies (Kularathnage et al. 2023; Senanayake et al. 2023; Du et al. 2024; Hongsanan et al. 2025) confirming that *Dyfrolomyces* and *Melomastia* are distinct genera.

#### ﻿ Melomastia beihaiensis

Taxon classificationFungiAscomycotaDothideomycetes

T.Y. Du, K.D. Hyde & Tibpromma, Fungal Diversity 122: 167 (2023)

E0D2D4AB-7617-51C0-A1FF-C90C4AD29189

Facesoffungi Number: FoF10262

Index Fungorum: IF558764

Fig. 7

##### Description.

***Saprobic*** on dead branch of *Volkameria inermis* L. **Sexual morph: *Ascomata*** (including neck) 300–450 μm high × 250–400 µm diam. (x̄ = 360 × 370 μm, n = 10), visible on the host surface as raised spots, dark brown to black, semi-immersed to immersed, solitary or aggregated, globose to subglobose, ostiolate, carbonaceous. ***Ostiolar canal*** 200–250 high μm × 100–150 µm diam. (x̄ = 220 × 120 μm, n = 10), cylindrical, straight, dark brown to black, periphyses absent. ***Peridium*** 45–60 µm diam. (x̄ = 52 μm, n = 15), comprising 3–5 inner layers of light brown cells of *textura prismatica*, several outer layers of dense, dark brown cells of *textura angularis* fused with host tissues. ***Hamathecium*** 2–3 µm wide, comprising hyaline, filiform, unbranched, aseptate pseudoparaphyses, longer than asci, attached at the base, embedded in a gelatinous matrix. ***Asci*** 140–200 × 6–8 µm (x̄ = 160 × 7 μm, n = 20), 8-spored, bitunicate, cylindrical, elongate, minute pedicellate, rounded at the apex. ***Ascospores*** 18–32 × 6–8 µm (x̄ = 26 × 7 μm, n = 30), hyaline, overlapping, uni-seriate, fusiform to broadly fusiform, conical at upper part and the lower end is truncate, distinct 3-septate at maturity, one large guttule in each cell, thin walled. **Asexual morph**: Undetermined.

**Figure 7.**
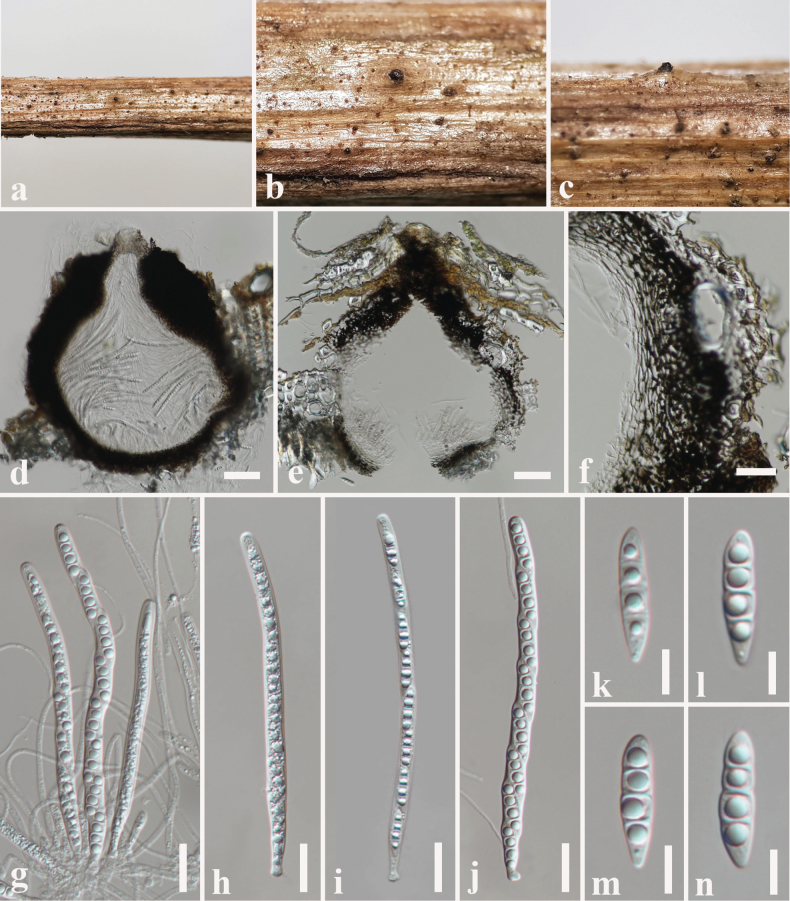
*Melomastia beihaiensis* (SZU 25-036, new host record). a. The plant substrate; b, c. Appearance of ascomata on substrate; d, e. Vertical sections through ascomata; f. Peridium; g. Pseudoparaphyses with asci; h–j. Asci; k–n. Ascospores. Scale bars: 50 μm (d, e); 20 μm (f–j); 10 μm (k–n).

##### Culture characteristics.

Colonies on PDA reaching 35 mm diameter after 1 week at 25 °C, colonies from above: circular, margin undulate, dense, flat, olive green at the margin, white in the centre; reverse: dark olive green at the margin, grey in the centre.

##### Material examined.

China • Guangdong Province, Shenzhen, on dead branch of *Volkameria inermis* L. (Lamiaceae), 11 June 2025, N. I de Silva NSZU6 (SZU 25-036), living culture, MBSZU 25-048.

##### Notes.

*Melomastia beihaiensis* was identified from dead stems of *Chromolaena odorata* (Asteraceae) in Beihai, Guangxi Province, China (Senanayake et al. 2023). The morphological characteristics of our collection (SZU 25-036) resembles *M. beihaiensis* (HKAS 121125) in having semi-immersed to immersed, globose to subglobose, ostiolate, carbonaceous ascomata, cylindrical, elongate, asci with minute pedicel and hyaline, fusiform to broadly fusiform, 3-septate, guttulate ascospores (Senanayake et al. 2023). We noted overlapping size ranges for asci and ascospores between the new strain (SZU 25-036) and the holotype of *M. beihaiensis* (HKAS 121125). The holotype possesses (125–)135–190 × 5–7 µm asci and 16–28 × 5–7 µm ascospores (Senanayake et al. 2023), whereas the new collection (SZU25-036) exhibits 140–200 × 6–8 µm asci and 18–32 × 6–8 µm ascospores. Multi-gene phylogeny also indicates that our collection (SZU25-036) nested with *M. beihaiensis* (KUMCC 21-0084) with 83% ML, 0.95 BYPP support (Fig. 4). *Melomastia beihaiensis* (MBSZU 25-048), displays a red colour in the pairwise identity matrix, indicating 97–98% identity with the ex-type strain of *M. beihaiensis* (KUMCC 21-0084) (Fig. 4a, b). Therefore, based on morphological and phylogenetic evidence, we report our collection as the first host record of *M. beihaiensis* on a dead branch of *Volkameria inermis* (Lamiaceae).

#### ﻿ Melomastia hydei

Taxon classificationFungiAscomycotaDothideomycetes

Hongsanan, Khuna & Xie N., Mycosphere 16(2): 48 (2025)

336A8036-1C7B-5452-8760-09DD14C72D19

Index Fungorum: IF903733

Facesoffungi Number: FoF17754

Fig. 8

##### Description.

***Saprobic*** on dead branch of *Acanthus ilicifolius* L. **Sexual morph: *Ascomata*** (including neck) 450–655 μm high × 400–560 µm diam. (x̄ = 550 × 490 μm, n = 10), black, visible as cone-shaped structures on host surface, solitary, scattered to gregarious, immersed to semi-immersed, globose to subglobose, coriaceous to carbonaceous, ostiolate. ***Ostiolar canal*** 170–210 μm high × 130–150 µm diam. (x̄ = 190 × 145 μm, n = 10), central, dark brown to black, ostiolar canal internally covered by filiform periphyses. ***Peridium*** 45–60 µm diam. (x̄ = 52 μm, n = 15), thick-walled, comprising 5–7 inner layers of light brown cells of *textura angularis*, several outer layers of dense, dark brown cells of *textura angularis* fused with host tissues. ***Hamathecium*** 1–2 μm wide, comprising hyaline, filiform, aseptate, unbranched pseudoparaphyses, longer than asci, embedded in a gelatinous matrix. ***Asci*** 135–160 × 5–7 µm (x̄ = 154 × 6 μm, n = 20), 8-spored, bitunicate, cylindrical, straight or slightly curved, rounded apex, with short pedicel. ***Ascospores*** 19–22 × 5–7 µm (x̄ = 20 × 6 μm, n = 30), hyaline, overlapping, uni-seriate, fusiform, straight or slightly curved, tapering towards both ends, smooth-walled, 3-septate, guttules in each cell, without a sheath or appendages. **Asexual morph**: Undetermined.

**Figure 8.**
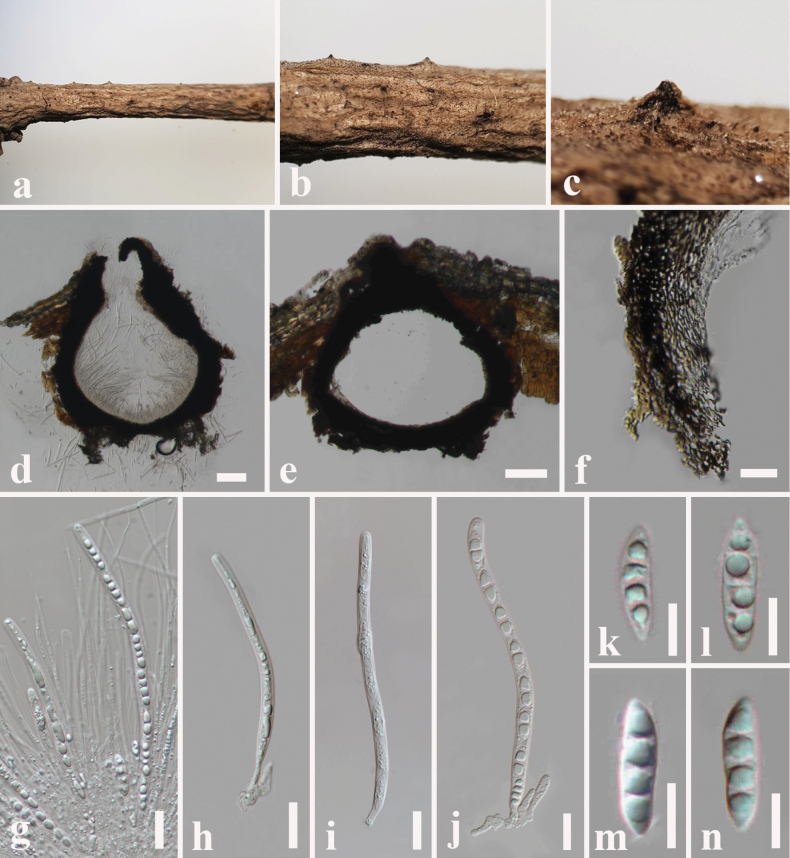
*Melomastia hydei* (SZU 25-037, new host record). a. The plant substrate; b, c. Appearance of ascomata on substrate; d, e. Vertical sections through ascomata; f. Peridium; g. Pseudoparaphyses with asci; h–j. Asci; k–n. Ascospores. Scale bars: 100 μm (d, e); 20 μm (f–j); 10 μm (k–n).

##### Culture characteristics.

Colonies on PDA reaching 32 mm diameter after 1 week at 25 °C, colonies from above: circular, margin entire, dense, flat, white at the margin, pale brown in the centre; reverse: white at the margin, pale brown in the centre.

##### Material examined.

China • Guangdong Province, Shenzhen, on dead branches of *Acanthus ilicifolius* L. (Acanthaceae), 11 June 2025, N. I de Silva NSZU13 (SZU 25-037), living culture, MBSZU 25-049.

##### Notes.

Our collection (SZU 25-037) morphologically fits well with the holotype of *Melomastia hydei* (SZU 25–003) indicating similar characteristics including globose to subglobose, ostiolate ascomata, 8-spored, bitunicate, cylindrical asci and hyaline, fusiform to broadly fusiform, 3-septate, guttulate ascospores (Hongsanan et al. 2025). The phylogenetic analysis revealed that the new strain (MBSZU 25-049) clustered with the ex-type of *M. hydei* (MBSZU 25–003) with 77% ML and 1.00 BYPP) (Fig. 4). The new strain, *M. hydei* (MBSZU 25-049), denoted ‘red’ in the colour-coded matrix with *M. hydei* strains (MBSZU 25-003, MBSZU 25-004), indicating the highest identity scores (96–97%) and confirming their close relatedness (Fig. 4a, b). The type of *M. hydei* was found on decaying twigs of Beach Naupaka (*Scaevola taccada*, Goodeniaceae) in Guangdong Province, China (Hongsanan et al. 2025). We herein provide a new host record for this species, based on the collection from dead branches of *Acanthus ilicifolius* (Acanthaceae) in Guangdong Province, China.

#### ﻿ Melomastia fusispora

Taxon classificationFungiAscomycotaDothideomycetes

W.L. Li, Maharachch. & Jian K. Liu, J. Fungi 8 (1, no. 76): 7 (2022)

324ED68B-5C3D-571F-AAD9-EDA9DACE1BED

Index Fungorum: IF841499

Facesoffungi Number: FoF10533

Fig. 9

##### Description.

***Saprobic*** on dead twigs of *Ficus* species. **Sexual morph: *Ascomata*** (including neck) 620–700 μm high × 610–650 µm diam. (x̄ = 670 × 640 μm, n = 10), black, solitary, gregarious, visible as cone-shaped structures on the host surface, immersed to semi-immersed, erumpent through host tissue, globose to subglobose, coriaceous to carbonaceous, ostiolate. ***Ostiolar canal*** 240–280 μm high × 170–240 µm diam. (x̄ = 260 × 210 μm, n = 10), central, dark brown to black, carbonaceous, ostiolar canal internally covered by filiform periphyses. ***Peridium*** 60–75 µm diam. (x̄ = 68 μm, n = 15), two-layered, comprising 4–6 inner layers of light brown cells of *textura angularis*, several outer layers of dense, dark brown cells of *textura angularis* fused with host tissues. ***Hamathecium*** 1–2 μm wide, comprising dense, hyaline, filiform, aseptate, unbranched pseudoparaphyses. ***Asci*** 150–210 × 6–8 µm (x̄ = 178 × 7 μm, n = 20), 8-spored, bitunicate, cylindrical, elongate, slightly flexuous, apically round with ocular chamber, short pedicel. ***Ascospores*** 18–30 × 6–7.5 µm (x̄ = 26 × 7.1 μm, n = 30), hyaline, overlapping, uni-seriate, fusiform, with rounded to acute ends, narrow towards apex, 3-septate, constricted at the central septum, with guttules in each cell. **Asexual morph**: Undetermined.

**Figure 9.**
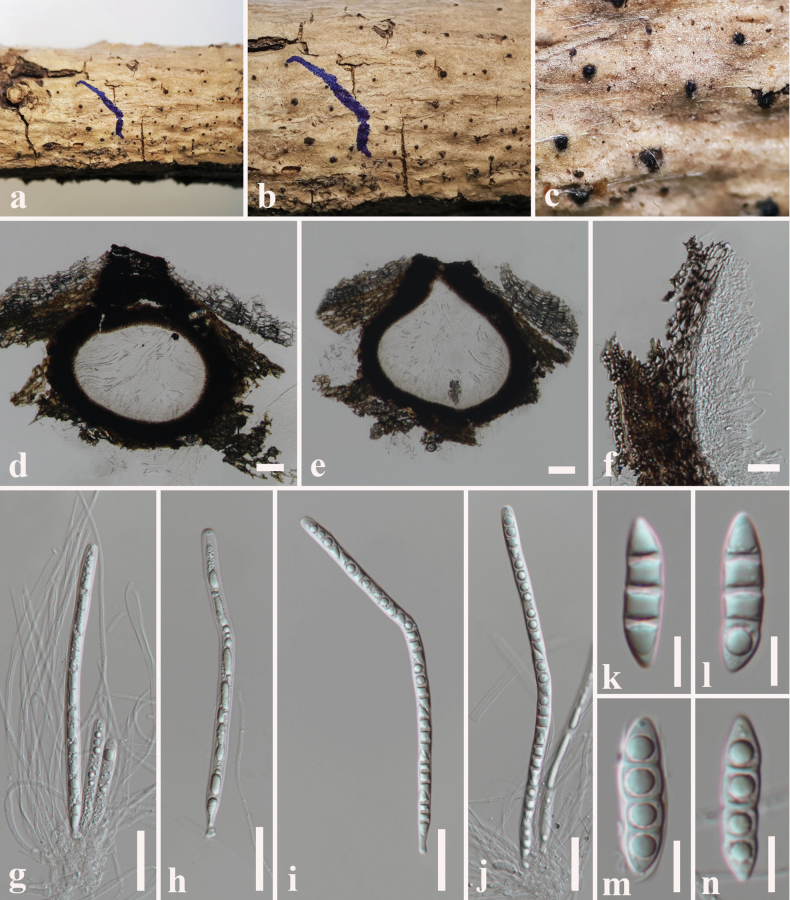
*Melomastia fusispora* (SZU 25-038, new host record). a. The plant substrate; b, c. Appearance of ascomata on substrate; d, e. Vertical sections through ascomata; f. Peridium; g. Pseudoparaphyses with asci; h–j. Asci; k–n. Ascospores. Scale bars: 100 μm (d, e); 20 μm (f); 30 μm (g–j); 10 μm (k–n).

##### Culture characteristics.

Colonies on PDA reaching 35 mm diameter after 1 week at 25 °C, colonies from above: circular, margin entire, dense, flat, cream at the margin, pale brown in the centre with white mycelia; reverse: white at the margin, brown in the centre.

##### Material examined.

China • Guangdong Province, Shenzhen, on dead twigs of *Ficus* sp. (Moraceae), 11 June 2025, N. I de Silva SZ15 (SZU 25-038), living culture, MBSZU 25-050.

##### Notes.

*Melomastia fusispora* was first reported from a dead branch of *Olea europaea* (Oleaceae) in Sichuan Province, China (Li et al. 2022). We recovered this species from dead twigs of *Ficus* sp. (Moraceae), in Guangdong Province, China. Our collection (SZU 25-038) morphologically fits well with *M. fusispora* (HKAS 121316) and the dimensions of asci (150–210 × 6–8 μm vs. 200–231 × 7.6–9.2 μm) and ascosproes (18–30 × 6–7.5 μm vs. 27.5–32 × 6.5–7.5 μm) are also comparable with the holotype (Li et al. 2022). The new isolate MBSZU 25-050 clustered with ex-type *M. fusispora* (CGMCC3.20618) in multi-gene phylogeny (LSU, SSU, ITS and *tef1-a*). The comparison of nucleotide substitutions between our strain (MBSZU 25-050) and the ex-type strain of *M. fusispora* (CGMCC3.20618) showed 0.78% (5/640) and 1.02% (8/779) differences (without gaps) in the ITS and *tef1-α* genes, respectively. Pairwise identity scores of 98% were calculated for the new strain *M. fusispora* (MBSZU 25-050) in comparisons with *M. fusispora* (CGMCC 3.20618, UESTCC 21.0001) (Fig. 4b). Therefore, based on morphology and phylogeny, a new host record for *M. fusispora* is provided.

#### ﻿ Melomastia shenzhenensis

Taxon classificationFungiAscomycotaDothideomycetes

N.I. de Silva, Tennakoon, S. Hongsanan sp. nov.

A019E67E-1F00-5482-9749-38AB08D52D44

Index Fungorum: IF904327

Facesoffungi Number: FoF18099

Fig. 10

##### Etymology.

The epithet “*shenzhenensis*” refers to the habitat ‘Shenzhen’, where the holotype was collected.

**Figure 10.**
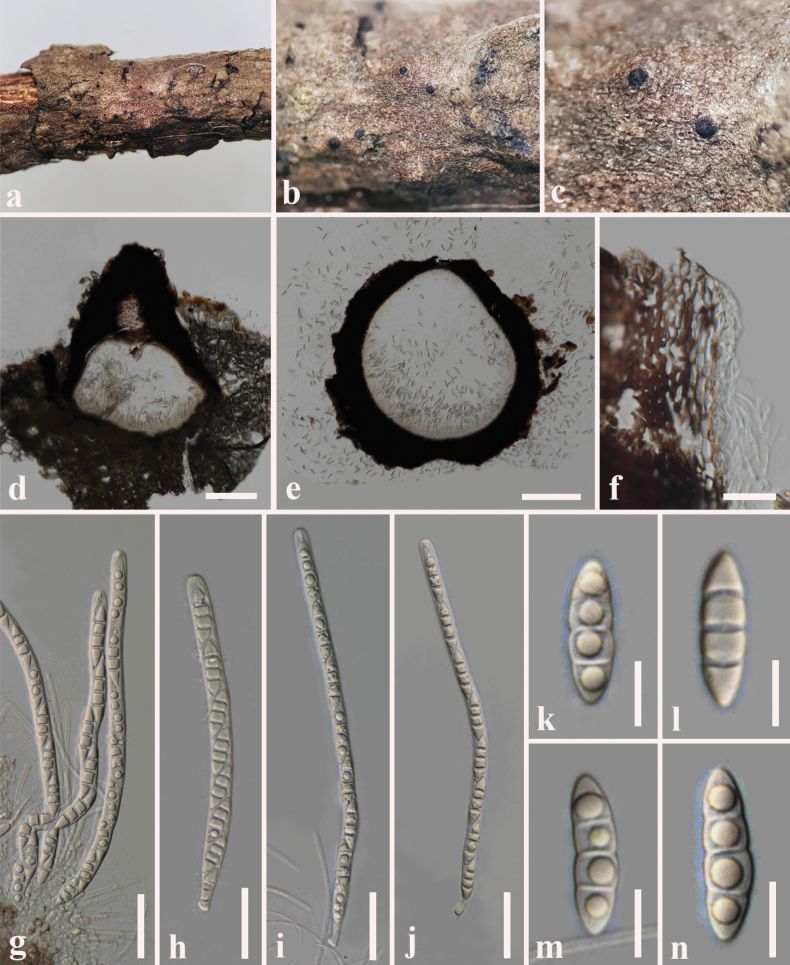
*Melomastia shenzhenensis* (SZU 25-039, holotype). a. The plant substrate; b, c. Appearance of ascomata on substrate; d, e. Vertical sections through ascomata; f. Peridium; g. Pseudoparaphyses with asci; h–j. Asci; k–n. Ascospores. Scale bars: 200 μm (d, e); 20 μm (f); 30 μm (g–j); 10μm (k–n).

##### Holotype.

SZU25-039.

##### Description.

***Saprobic*** on dead twigs of *Ficus* species. **Sexual morph: *Ascomata*** (including neck) 650–780 μm high × 560–670 µm diam. (x̄ = 720 × 590 μm, n = 10), dark brown, black, solitary or gregarious, erumpent to superficial when mature, globose to subglobose, coriaceous, papillate, ostiolate. ***Ostiolar canal*** 270–320 μm high × 190–260 µm diam. (x̄ = 310 × 230 μm, n = 10), central, dark brown to black, conical, carbonaceous, internally covered by filiform periphyses. ***Peridium*** 70–85 µm diam. (x̄ = 78 μm, n = 15), two-layered, comprising 5–6 inner layers of light brown cells of *textura angularis*, several outer layers of thick, dark brown cells of *textura angularis* fused with host tissues. ***Hamathecium*** 1–2 μm wide, comprising dense, hyaline, filiform, aseptate, unbranched pseudoparaphyses. ***Asci*** 145–180 × 6–8 µm (x̄ = 168 × 7 μm, n = 20), 8-spored, bitunicate, fissitunicate, cylindrical, straight or slightly curved, apically round, with ocular chamber, short pedicellate. ***Ascospores*** 19–23 × 4–5.8 µm (x̄ = 21 × 5.3 μm, n = 30), hyaline, overlapping, uni-seriate, fusiform with acute ends, 3-septate, not constricted at the septa, smooth-walled, guttules in each cell. **Asexual morph**: Undetermined.

##### Culture characteristics.

Colonies on PDA reaching 28 mm diameter after 1 week at 25 °C, colonies from above: circular, margin entire, dense, flat, cream at the margin, pale olive-green in the centre; reverse: white at the margin, olive-green in the centre.

##### Material examined.

China • Guangdong Province, Shenzhen, on dead branches of *Ficus* sp. (Moraceae), 11 June 2025, N. I de Silva NSZU3 (SZU 25-039, holotype), ex-type living culture, MBSZU 25-051.

##### Notes.

A new strain (MBSZU 25-051) isolated from dead branches of *Ficus* sp. (Moraceae), shows high statistical support value (ML/BYPP = 100%/1.00) and closely clustered with *Melomastia oleae* (CGMCC 3.20619 and UESTCC 21.0003). The new strain (MBSZU 25-051) indicates the highest pairwise identity value (94.3%, represented by pink) with two strains of *M. oleae* strains i.e. CGMCC 3.20619 (ex-type) and UESTCC 21.0003 (Fig. 4a, b). Aside from the two *M. oleae* strains, the remaining taxa display 78–94% pairwise identity scores with *M. shenzhenensis* (MBSZU 25-051), corresponding to the green to olive-green colour range in the matrix. Morphologically, the new collection (SZU 25-039) differs from its closest *M. oleae* by larger ascomata (650–780 μm high × 560–670 µm diam. vs. 410–440 μm × 493–520 µm) and ostiolar canal (270–320 μm high × 190–260 µm diam. vs. 20.5–50 × 60–83 µm) (Li et al. 2022). The peridium of the new collection (SZU25-039) (70–85 µm) is larger than *M. oleae* (54–65 µm) (Li et al. 2022). Further, the new collection (SZU25-039) was also distinguished from *M. oleae* in having smaller asci (145–180 × 6–8 µm vs. 209–237 × 7.5–9 µm) and ascospores (19–23 × 4–5.8 µm vs. 28–34 × 6–7 μm) (Li et al. 2022). Thus, we introduce *M. shenzhenensis* here as a new species in genus *Melomastia*.

## ﻿Discussion

In this study, we introduce a new species, *Dictyosporium thecatum* on a dead branch of *Volkameria inermis* L. (Lamiaceae) in the coastal region of China. Most accepted *Dictyosporium* species are represented by asexual morphs (Yang et al. 2018; Hyde et al. 2020; Liu et al. 2023; Tennakoon et al. 2023; Shu et al. 2024; Wang et al. 2025), except for *D. sexualis* (Boonmee et al. 2016), *D. meiosporum* (Liu et al. 2015) from Thailand, *D. karsti* (Zhang et al. 2023) and *D. thecatum* described in the present study from China. Phylogenetically, *D. thecatum* is sister to *D. sexualis*. Furthermore, *Dictyosporium karsti* forms a separate sub-clade with *D. olivaceosporum* (KH 375) that is closely related to the group consisting *D. thecatum* and *D. sexualis*. However, *D. meiosporum* (MFLUCC 10-0131) is distantly related to *D. thecatum* and clustered with *D. tetrasporum* (KT 265) (Fig. 1). Amongst the three sexual morph taxa considered (*D. karsti*, *D. meiosporum* and *D. sexualis*), the new species *D. thecatum* (MBSZU 25-047) is most closely related to *D. sexualis* (MFLUCC 10-0127), with which it shares the highest pairwise identity (95%). It shows lower identity values (92–93%) with *D. karsti* and *D. meiosporum* (Fig. 2b). These four sexual morph taxa share similar morphology in having black, superficial, solitary or scattered, globose to sub-globose, ostiolate ascomata, brown to black peridium composed of cells of *textura angularis*, 8-spored, bitunicate, fissitunicate, clavate to cylindrical asci, hyaline, elongated-ellipsoid, clavate, 1-septate, ascospores surrounded by a mucilaginous sheath (Liu et al. 2015; Boonmee et al. 2016; Zhang et al. 2023). *Dictyosporium thecatum* is distinguished from *D. karsti*, *D. meiosporum* and *D. sexualis* by its smaller asci and ascospores. Larger asci and ascospore sizes are reported for three species: in *D. sexualis* (100–145 × 10–14 μm/36–48 × 6–8 μm (asci/ascospores); Boonmee et al. (2016)), *D. karsti* (97 × 13.8 μm/30.8 × 5 μm; Zhang et al. (2023)) and *D. meiosporum* (83–135.5 × 13–17 μm/31–39 × 6–8.5 μm; Liu et al. (2015)). Furthermore, *Dictyosporium thecatum* is differentiated from *D. karsti*, *D. meiosporum* and *D. sexualis* by a distinct, 5–7 μm thick mucilaginous sheath surrounding its ascospores (Liu et al. 2015; Boonmee et al. 2016; Zhang et al. 2023). In this study, we observed brown to black, compact, sporodochia (asexual morph) of *Dictyosporium thecatum* in the culture (PDA media). Similarly, *D. meiosporum* produced conidia in the culture (Liu et al. 2015). In contrast, the asexual morph was not reported from *D. karsti* and *D. sexualis* (Boonmee et al. 2016; Zhang et al. 2023). The morphological variations possessed by the new species, *Dictyosporium thecatum*, may enhance its survival in coastal environments. Therefore, future investigations in different habitats should reveal novel species with unique adaptive morphologies.

The current study introduced a novel *Melomastia* species, *M. shenzhenensis* and identified three new host associations of *M. fusispora*, *M. hydei* and *M. beihaiensis*. *Melomastia* species show a cosmopolitan distribution in both temperate and tropical countries, i.e. Africa (Central African Republic, Ivory Coast, South Africa), Asia (Brunei, China, India: Andaman and Nicobar Islands, Iran, Japan, Kazakhstan, Kirgizstan, Malaysia, Philippines, Thailand, Turkmenistan), Australia, Europe (Czechia, France, Germany, Italy) and South America (Argentina, Brazil, Chile) (Li et al. 2022; Kularathnage et al. 2023; Farr and Rossman 2025). Amongst these countries, several *Melomastia* species have recently been reported from China. *Melomastia maolanensis* was identified from dead plant substrates in Guizhou Province (initially described as ‘*Dyfrolomyces maolanensis*’) (Norphanphoun et al. 2017; Zhang et al. 2017). Subsequently, 16 novel *Melomastia* species and several new host/geographical records have been identified in China at an accelerated rate over the past three years (since 2022), reported from Guangxi, Guizhou, Guangdong, Sichuan and Yunnan Provinces on various host plants. Four species, namely, *M. fusispora*, *M. oleae*, *M. sichuanensis* and *M. winteri* were introduced by Li et al. (2022) from the dead branches of *Olea europaea* (Oleaceae) in Sichuan Province in China. In addition, several novel taxa have been established: in Guangxi Province, *M. beihaiensis* on dead stems of *Chromolaena odorata* (Senanayake et al. 2023); in Guizhou Province, *M. chinensis* on dead wood (Habib et al. 2025); and in Yunnan Province, *M. diqingensis* on dead twigs of *Rhododendron rubiginosum* (Ren et al. 2024) and *M. puerensis* on dead branches of *Hevea brasiliensis* (Xu et al. 2024). Another research team investigated saprobic fungi associated with *Aquilaria* spp., an important agarwood resin-producing tree. Their work discovered four novel species, *M. aquilariae*, *M. guangdongensis*, *M. maomingensis* and *M. yunnanensis* and also reported a new host and geographical record for *M. sinensis* in Guangdong and Yunnan Provinces, China (Du et al. 2024; Manawasinghe et al. 2024). Moreover, three species have been isolated in Guangdong Province, China: *M. hydei* on decaying twigs of *Scaevola taccada* (Hongsanan et al. 2025), *M. loropetalicola* on dead stems of *Loropetalum chinense* (Dong et al. 2023) and *M. pyriformis* on decaying wood (Kularathnage et al. 2023). In this study, we add one new species, *M. shenzhenensis* and report three new host associations for *M. fusispora*, *M. hydei* and *M. beihaiensis* in the same province.

The Sequence Demarcation Tool version 1.2 (SDT v.1.2), a free user-friendly computer programme provides a robust and highly reproducible means of pairwise genetic identity calculations to classify any set of nucleotide or amino acid sequences (Muhire et al. 2014). This computational tool aligns every possible pair of sequences using one of three optional alignment algorithms, namely, MUSCLE, ClustalW2 or MAFFT and, subsequently, calculates a Needleman-Wunsch (NW) pairwise alignment-based genetic identity score for each pair (Muhire et al. 2014). The resulting score can be retrieved as a numerical matrix. In addition, it depicts colour-coded pairwise-identity matrix with according pairwise identity values. To cluster pairwise identities in an evolutionarily meaningful way, the sequences are ordered along the matrix axes according to their placement in the Maximum Likelihood (ML) phylogram. Thus, it allows us to detect newly-obtained sequences and their phylogenetically closely-related taxa easily and corresponding pairwise identity scores according to the colours of the cells in the matrix. The colour-coded pairwise identity matrix and its corresponding numerical data matrix further validate the phylogenetic analyses and species delimitation. The colour-coded plot facilitates the easy identification of taxa with high similarity values and distinguishes those with lower similarity to the strains of interest. Therefore, pairwise identity calculations generated using the SDT application offer additional evidence for species identification.

## Supplementary Material

XML Treatment for Dictyosporium thecatum

XML Treatment for Melomastia beihaiensis

XML Treatment for Melomastia hydei

XML Treatment for Melomastia fusispora

XML Treatment for Melomastia shenzhenensis

## ﻿Additional information

### Use of AI

No use of AI was reported.

### Author ORCIDs

Nimali I de Silva https://orcid.org/0000-0002-1577-280X

Danushka S. Tennakoon https://orcid.org/0000-0003-2306-1255

Ning Xie https://orcid.org/0000-0002-5866-8535

Sinang Hongsanan https://orcid.org/0000-0003-0550-3152

### Data availability

All of the data that support the findings of this study are available in the main text.
